# Effect of Didactic Training on Barriers and Biases to Treatment of Opioid Use Disorder: Meeting the Ongoing Needs of Patients with Opioid Use Disorder in the Emergency Department during the COVID-19 Pandemic

**DOI:** 10.3390/healthcare10122393

**Published:** 2022-11-29

**Authors:** Emily Johnson, Maria Bolshakova, Aidan Vosooghi, Chun Nok Lam, Rebecca Trotzky-Sirr, Ricky Bluthenthal, Todd Schneberk

**Affiliations:** 1 Department of Emergency Medicine, Keck School of Medicine, University of Southern California, Los Angeles, CA 90033, USA; 2 Department of Population and Public Health Sciences, Keck School of Medicine, University of Southern California, Los Angeles, CA 90032, USA; 3 Keck School of Medicine, University of Southern California, Los Angeles, CA 90033, USA; 4 Addiction Medicine, University of Southern California Medical Center, Los Angeles, CA 90033, USA

**Keywords:** opioid use disorder, medication for addiction treatment, buprenorphine, emergency medicine, graduate medical education

## Abstract

In the wake of COVID-19, morbidity and mortality due to Opioid Use Disorder (OUD) is beginning to emerge as a second wave of deaths of despair. Medication assisted treatment (MAT) for opioid use disorder MAT delivered by Emergency Medicine (EM) providers can decrease mortality due to OUD; however, there are numerous cited barriers to MAT delivery. We examined the impact of MAT training on these barriers among EM residents in an urban, tertiary care facility with a large EM residency. Training included the scripted and standardized content from the Provider Clinical Support System curriculum. Residents completed pre- and post-training surveys on knowledge, barriers, and biases surrounding OUD. We performed Wilcoxon matched-pairs signed-ranks test to detect statistical differences. Of 74 residents, 49 (66%) completed the pre-training survey, and 34 (69%) of these completed the follow-up survey. Residents reported improved preparedness to treat aspects of OUD across all areas queried, reported decreased perception of barriers to providing MAT, and increased comfort prescribing naloxone, counseling patients, prescribing buprenorphine, and treating opioid withdrawal. A didactic training on MAT was associated with residents reporting improved comfort providing buprenorphine and naloxone. As the wake of morbidity and mortality from both COVID and OUD continue to increase, programs should offer dedicated training on MAT.

## 1. Introduction

Opioid-related overdose death rates have been steadily climbing over the past decade, but the COVID-19 pandemic, along with the domination of fentanyl in the drug supply has led to unprecedented highs, with a record 80,590 opioid overdose deaths in 2021 [1,2,3]. Despite a decrease in all-cause overall emergency department (ED) visits in 2020 (compared to 2018–2019), opioid-related overdose visits increased by 28.5% across six US healthcare systems in 25 states amidst the COVID-19 pandemic [1,2,3]. Emergency departments frequently encounter patients with opioid use disorder (OUD), which provides EDs with a unique position to offer evidence-based medication for addiction treatment (MAT) [4,5,6]. The interaction of the severity of the opioid crisis with the pandemic’s effects on people who use drugs such as social isolation, homelessness, stigma, and marginalization creates a critical need to address OUD during ED visits [7,8].

Emergency departments have increasingly embraced their role as a low barrier source of MAT initiation, specifically using buprenorphine to treat opioid withdrawal symptoms [9]. Treatment of OUD with buprenorphine in the ED may help build a rapport with patients, leading to meaningful discussion of treatment goals and MAT 1-month maintenance rates as high as 50% [10,11]. Despite the growing national attention on OUD and the effectiveness of MAT, studies find emergency medicine (EM) physicians are generally in favor of using buprenorphine, yet don’t feel comfortable or ready to initiate it themselves [12,13,14]. Physicians cite lack of confidence, knowledge, and competing ED needs as barriers; and increased training opportunities, institutional support, department protocols, and feedback of patient experiences as facilitators [12,13]. Prior to 2020, all physicians had to complete an 8 h training to obtain an ‘X-Waiver’ to be able to prescribe buprenorphine, another notable barrier that contributed to only 4% of licensed physicians being approved to prescribe [15]. Additionally, we know provider stigma and bias can impact the care of patients with OUD [16]. This stigma can deter patients with substance use disorders from seeking addiction treatment and routine medical care, which puts patients at significant risk, especially in a time of overlapping opioid and COVID pandemics, where access to care is strained [7].

EM residents are more eager to initiate MAT than attendings, and most consider it very important to have training in buprenorphine initiation [17,18]. Nonetheless, the role of formal training for EM residents in recognizing, administering, and prescribing buprenorphine has not been established and few programs have developed focused curricula on addiction medicine [19]. Only one in four residency programs in internal medicine, psychiatry, and family medicine provided 12 or more hours of substance use education leading to residents feeling unprepared to treat or discuss substance use with their patients [20,21]. Residents report lack of knowledge and training on treating OUD as a major barrier to prescribing MAT [22]. While the majority of EM residents were interested in receiving training necessary to prescribe buprenorphine, only half of respondents thought they would receive the training during their residency [18].

Given the persistent stigma of drug use and factors such as housing instability or difficulty accessing transportation that may limit many patient’s ability to effectively access care, the Emergency Department’s role as an accessible safety net for obtaining OUD treatment is clear [10]. We implemented a brief didactic MAT training in an EM residency program and examined pre-post changes in perceived barriers to offering medication for OUD and statements of bias towards patients with OUD among EM residents.

## 2. Materials and Methods

LAC + USC is an urban, safety net, tertiary care facility with a large emergency medicine residency. We invited an addiction medicine physician to conduct MAT training for all EM residents in January 2020 during protected conference time. Residents must attend 70% of conferences per year. The training was directed by the addiction medicine physician and utilized a condensed version of the X-waiver scripted content from the Provider Clinical Support System (PCSS) for a total of four hours of training. The content included brief overviews of all sections covered in the core X waiver training including those on medication pharmacology, evidence-based counselling, and neurobiology, but with a more in depth focus on practical case-based content on recognition of substance use disorders, treatment modalities, efficacy of MAT, and buprenorphine induction in the ED setting. In addition to the board-certified addiction medicine specialist leading the training, X-waivered Emergency Physicians (authors T.S. and E.J.) were present to respond to questions on medication delivery in the ED setting. The training also covered connection to outpatient care resources in the area. Prior to training, we surveyed residents on preparedness, barriers, and biases to treating patients with OUD.

Residents were informed of the upcoming training by resident and faculty champions (authors E.J. and T.S.) and invited to participate in a research study prior to the lecture with a USD 5 gift card for participation. Surveys were completed anonymously with a self-created coded identifier (first two letters of make of first car, month of birth, etc.). The surveys could be completed on mobile phones or computers, and residents were reminded of the opportunity to complete the survey prior to a break between didactic sessions during protected conference time. Three months after training, in March 2020, all residents were invited to participate in a post-survey containing the same questions regarding comfort with buprenorphine and biases surrounding OUD. The survey deployed included an abbreviated version of that used by Lowenstein et al. describing barriers to buprenorphine delivery among ED providers, and the Medical Condition Regard Scale [23,24]. Specific topics included barriers to deploying MAT in the ED, stigma with treating patients with OUD and competency with addiction related care. See Appendix A for sample questions and response options. Residents could skip any question. Responses were matched using the self-produced coded identifier. We calculated the median score and interquartile range (IQR), and used Wilcoxon matched-pairs signed-ranks test to compare pre- and post-training responses. The Wilcoxon test does not presume normality of ordinal responses. To address the concern of multiple comparisons within each item set, we computed the sharpened false discovery rate (FDR) *q*-values to account for the expected proportion of rejections that are due to type I error [25]. A sharpened FDR *q*-value of <0.05 is considered statistically significant. Analyses were performed in STATA. The study was reviewed and classified as exempt by the University of Southern California Institutional Review Board.

## 3. Results

Of the 74 residents invited to participate, 51 residents attended the MAT training (69%). There were 49 (66%) who completed the first survey and 37 (50%) who completed the second survey, with 34/49 (69%) matching codifiers for pre-post analysis. A spectrum of respondents completed the survey, from 22 to 25% per class year (PGY1–PGY4). Female respondents comprised the majority in both surveys (53% at baseline; 56% follow-up). In both pre- and post-survey, 51% of respondents were senior residents (PGY3 and PGY4).

### 3.1. Knowledge and Barriers

Table 1 illustrates the results of our pre- and post-survey (significance noted in bold at *q* < 0.05). There were high rates of self-reported preparedness (median score > 3, signaling “somewhat prepared” or “very prepared”) on the pre-training survey for screening (median: 4, IQR: 1) and diagnosis (median: 4, IQR: 1) of OUD as well as for counseling and prescribing naloxone (median: 4, IQR: 2 and median: 4, IQR: 3, respectively). Residents reported improved preparedness in the post-training survey across all knowledge areas queried, with the highest changes in feeling prepared to initiate buprenorphine treatment for opioid withdrawal (median: 3, IQR: 2 to median: 4, IQR: 1, *q* = 0.001), discussing medication options for OUD (median: 3, IQR: 2 to median: 4, IQR: 1, *q* = 0.001), calculating a Clinical Opioid Withdrawal Score (COWS) to assess acute withdrawal (median: 2, IQR: 2 to median: 4, IQR: 0, *q* = 0.001), and determining the level of care for patients presenting with OUD (median: 3, IQR: 2 to median: 4, IQR: 1, *q* = 0.001). The reported significance of some systems- and environment-specific barriers also decreased, including availability of social work/care coordination (median: 3, IQR: 3 to median: 1, IQR: 2., *q* = 0.003) and concerns about patient insurance and ability to follow up (median: 4, IQR: 4 to median: 1, IQR: 3, *q* = 0.022). Comfort counselling patients about buprenorphine as a barrier to MAT delivery also decreased (median: 4, IQR: 5 to median 1, IQR: 3, *q* = 0.022). Several systems factors did not reach statistical significance but had a *q* = 0.050, including lack of specialty support for complex problems (median: 1, IQR: 3 to median: 1, IQR: 1.5, *q* = 0.050), accessing protocols for buprenorphine (median: 3, IQR: 4 to median: 1, IQR: 2, *q* = 0.050), and financial concerns (median: 4, IQR: 5 to median: 1, IQR: 3.5, *q* = 0.050).

### 3.2. Bias

After training, residents were more likely to report that they felt comfortable counseling patients, prescribing buprenorphine, and treating opioid withdrawal (see Table 2).

### 3.3. Impact on Practice

We also performed an exploratory analysis to examine any correlation between training and buprenorphine prescribing and administration rates pre- and post-training. We saw marked increases in buprenorphine delivery for acute withdrawal in the ED and buprenorphine prescribing after the X-waiver intervention (see Supplementary Materials, Figure S1). The average prescription rate tripled in the six months prior to training to the six months following training (from 0.5 ± 0.12/1000 to 1.6 ± 0.56/1000 ED visits). Similarly, rates of administration of buprenorphine for acute withdrawal doubled (from 0.7 ± 0.16/1000 ED visits pre-training to 1.4 ± 0.34/1000 post-training). These increases in treatment observed for OUD patients persisted while adjusting for the marked decrease in ED volume during the start of the COVID-19 pandemic.

## 4. Discussion

A brief, one-time educational intervention in provision of treatment for OUD was associated with changes in responses by emergency medicine residents regarding medication for addiction treatment. At 3 months post-intervention, residents reported improved knowledge of MAT and decreased perceived impacts of both systems and personal barriers to prescribing harm reduction medications including both buprenorphine and naloxone. We find a practical training which enables resident physicians with tools to treat OUD may have contributed to increased comfort with MAT and allowed for application-based knowledge in EM residents.

With the removal of the mandated training hours necessary to obtain an X-Waiver to prescribe buprenorphine, residency programs may not feel the need to encourage focused education on MAT treatment. However, the problems surrounding the X-Waiver requirement seem to have stemmed from complexities of the entire waiver process and getting started rather than a lack of desire for the training itself [26]. The removal of the training requirement of the X-Waiver reduces significant structural barriers, but clinicians have repeatedly cited lack of knowledge and training as a fundamental barrier to MAT delivery [5,21]. Despite growing attention to OUD, removal of the X-Waiver training component did not mitigate the reduced growth of X-Waivered clinicians during the first two years of COVID-19 [27]. Conveniently, the X-Waiver curriculum has already been developed and can be integrated into residency training programs using dedicated didactics time, as we did here. As the COVID-19 pandemic has demonstrated, Emergency Medicine needs to recognize its role in caring for patients with OUD, which often intersect with other social vulnerabilities such as racial disparities, income inequality, and unstable housing. A more robust and informed EM workforce can be ready to meet this need through commitment to education on substance use disorder treatment in the acute setting, including medication assisted treatment delivery for OUD.

### Limitations

Surveys completed were anonymous to protect resident confidentiality; therefore, we could not address if the educational content delivered, and individual responses, were directly linked to resident prescribing patterns. This is a single-site study in an urban, diverse setting at a social safety net hospital, and may not generalize to other settings but could inform similar interventions within training centers. There may be selection bias accounting for some of the findings given that participation rates were moderate. We acknowledge the participation may have been influenced by presence of resident and faculty champions but hoped to ameliorate effects on individual responses through the anonymous electronic survey format. Due to the exploratory nature of this study and to facilitate replication, we chose to present the detailed survey items and their pre-post statistical comparisons. We computed the sharpened FDR *q*-values to address multiple comparisons in our data to account for inflated type I errors despite a small sample size. A larger sample population will provide greater statistical power to detect small, significant differences. Rates of prescribing and measurement of practice change were not designed to effectively limit the many confounders at play in large systems like the one described, and we present these data to lend veracity to the effect of the intervention but acknowledge the likely presence of other factors, such as the effect of experiential learning. There is also significant groundswell across medicine and emergency medicine in encouraging access to MAT that may have confounded our outcomes.

## 5. Conclusions

Although specific education is no longer required for buprenorphine prescribing, residency training education will still be necessary to enhance adoption of evidence-based addiction treatment in the ED. Our intervention demonstrated how low overhead training can lead to decreased perceived barriers for OUD treatment adoption among emerging generations of physicians as they develop lasting practice patterns [28,29,30,31]. The incorporation of training on medication for addiction treatment in residency curriculums is vital to equip emergency physicians to care for the many patients suffering in the ongoing opioid epidemic.

## Figures and Tables

**Table 1:** Pre-and Post-training Preparedness and Barriers to using Medication for OUD (*n* = 34).

The Next Questions Focus on Your Level of Preparation when Caring for Patients with Opioid Use Disorder. Thinking about Your Education, Training, and Work Experience, Please Describe How Prepared You Feel to: *	Pre Median (IQR)	Post Median (IQR)	*q*-Value
Screen for opioid use disorder	4 (1)	4 (1)	0.015
Diagnose opioid use disorder	4 (1)	4 (0)	0.005
Discuss medication options for treatment of opioid use disorder and withdrawal	3 (2)	4 (1)	0.001
Calculate a Clinical Opioid Withdrawal Scale (COWS) score for opioid withdrawal	2 (2)	4 (0)	0.001
Initiate buprenorphine for treatment of opioid withdrawal	3 (2)	4 (1)	0.001
Determine the appropriate level or care for patients with opioid use disorder (e.g., inpatient vs. outpatient treatment)	3 (2)	4 (1)	0.001
Connect patients from the ED to outpatient treatment	2 (2)	3 (2)	0.001
Counsel a patient about overdose prevention with naloxone	4 (2)	4 (0)	0.007
Prescribe naloxone for overdose prevention	4 (3)	4 (1)	0.001
**Please Indicate Whether or Not Each of the Following Are Barriers to Incorporating Buprenorphine into Your Practice for the Treatment of OUD:**	**Pre Median (IQR)**	**Post** **Median (IQR)**	***q*-Value**
Time constraints	4 (5)	3 (5)	0.697
Lack of available mental health or psychosocial support services	1 (2)	1 (2)	0.387
Concerns about diversion or misuse of medication	0 (2)	1 (2)	0.714
Attraction of drug users to your ED	0.5 (1)	1 (1)	0.714
Access to expert physician consultation	0 (1)	0 (1)	0.763
Lack of confidence in your ability to manage opioid use disorder	2 (4)	1 (4)	0.714
Availability of social work/care coordination support	3 (3)	1 (2)	0.003
Availability of referrals for substance use treatment services after discharge	5 (3)	5 (3)	0.815
Regulatory concerns related to prescribing buprenorphine	1 (2)	1 (1)	0.163
Lack of patient interest in treatment	0 (1)	0 (1)	0.697
Patient preference for other treatment	2 (3)	1.5 (3.5)	0.612
Lack of specialty backup for complex problems	1 (3)	1 (1.5)	0.050
Comfort with ordering buprenorphine	4 (4)	3 (4)	0.074
Resistance from your senior resident(s)	5 (3)	4 (3.5)	0.080
Access to protocols for initiating buprenorphine	3 (4)	1 (2)	0.050
Resistance from your attending	2 (3)	2 (3)	0.714
Concerns about medication safety or adverse effects	2 (2)	2 (2.5)	0.467
Financial concerns	4 (5)	1 (3.5)	0.050
Comfort with counselling patients about buprenorphine	4 (5)	1 (3)	0.022
Lack of patient need	1 (3)	1 (1.5)	0.169
Patient insurance, ability to follow up	4 (4)	1 (3)	0.022
Availability of nursing support	5 (3)	4 (3.5)	0.252

* Survey questions used with permission and adapted from a physician survey of barriers and facilitators for emergency department initiation of buprenorphine [23].

**Table 2:** Pre- and Post-Training Biases Around Patients with OUD (*n* = 34).

Please Answer the Following 11 Questions with Regard to Patients with Opioid Use Disorder (OUD) Presenting to the EMERGENCY DEPARTMENT ^±^	Pre Median (IQR)	Post Median (IQR)	*q*-Value
Working with patients like this is satisfying	54 (31)	60 (36)	0.254
Insurance plans should cover patients like this to the same degree that they cover patients with other conditions	100 (16)	85 (19)	0.177
There is little I can do to help patients like this	21 (33)	21 (26)	0.278
I feel especially compassionate towards patients like this	49.5 (34)	49.5 (34)	0.384
Patients like this irritate me	47 (52)	31.5 (37)	0.101
Treating patients like this is a waste of medical dollars	2 (10)	4 (9)	0.384
Patients like this are particularly difficult for me to work with	30.5 (52)	34 (39)	0.254
I can usually find something that helps patients like this feel better	62.5 (29)	62 (37)	0.337
I enjoy giving extra time to patients like this	51 (19)	44 (30)	0.177
I prefer not to work with patients like this	26 (34)	8.5 (32)	0.195
I find caring for patients with opioid use disorder as satisfying as my other clinical activities.	51 (33)	51.5 (26)	0.303
Patients with opioid use disorder are more challenging than the average patient to take care of	68 (24)	58 (36)	0.130
I feel comfortable treating opioid withdrawal in the Emergency Department	45 (48)	71.5 (33)	0.001
I feel comfortable treating patients with OUD with buprenorphine in the Emergency Department	50.5 (62)	72 (40)	0.031
I feel comfortable prescribing buprenorphine to patients with OUD	23.5 (57)	58.5 (37)	0.001
I often see patients with OUD in the ED	72.5 (47)	54.5 (46)	0.130
You need an “X” waiver to administer buprenorphine for acute withdrawal in the ED	2 (30)	2 (7)	0.177

^±^ Survey questions adapted from the Medical Condition Regard Scale [24]. *q*-value by Wilcoxon matched-pairs signed-ranks test, *q* < 0.05 is considered statistically significant [25].

**Figure A1:**
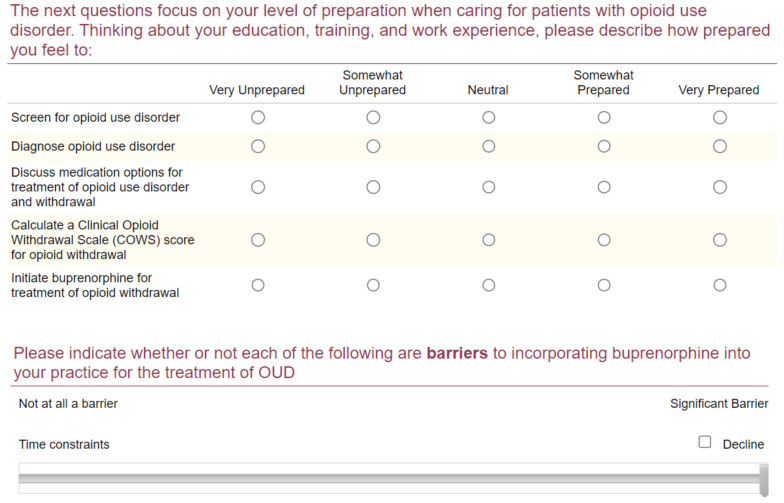
Sample of electronic survey questions and response options.

## Data Availability

Data is available by emailing the corresponding author.

## Supplementary Materials

The following supporting information can be downloaded at: https://www.mdpi.com/article/10.3390/healthcare10122393/s1, Figure S1: Medication for Opioid Use Disorder Delivery Pre- and Post-Training (*n* = 159,606 ED visits).

## Appendix A. Survey Question Format

The overall prompt stated “The next questions focus on your level of preparation when caring for patients with opioid use disorder. Thinking about your education, training, and work experience, please describe how prepared you feel to:” Responses used a 5-point scale ranging from Very Unprepared (1) to Very Prepared (5). The next question prompt examined barriers to prescribing with the statement “Please indicate whether or not each of the following are barriers to incorporating buprenorphine into your practice for the treatment of OUD:” and utilized a slider bar ranging from “not at all a barrier” (0) to “significant barrier” (7). Additionally, we deployed the Medical Condition Regard Scale to capture level of agreement with statements of biases around patients with OUD [24]. The prompt stated, “Please answer the following 11 questions with regard to patients with opioid use disorder (OUD) presenting to the Emergency Department” and used a slider bar from strongly disagree (0) to strongly agree (100). Residents could skip any question. See figure below:
