# Supplementary material for: Effect of Didactic Training on Barriers and Biases to Treatment of Opioid Use Disorder: Meeting the Ongoing Needs of Patients with Opioid Use Disorder in the Emergency Department during the COVID-19 Pandemic
Source: Healthcare (Basel). 2022 Nov 29;10(12):2393. doi: 10.3390/healthcare10122393 (PMC9778275; doi:10.3390/healthcare10122393)

Supplement: Figure S1. Medication for Opioid Use Disorder Delivery Pre- and Post-Training (n=159,606 ED visits)

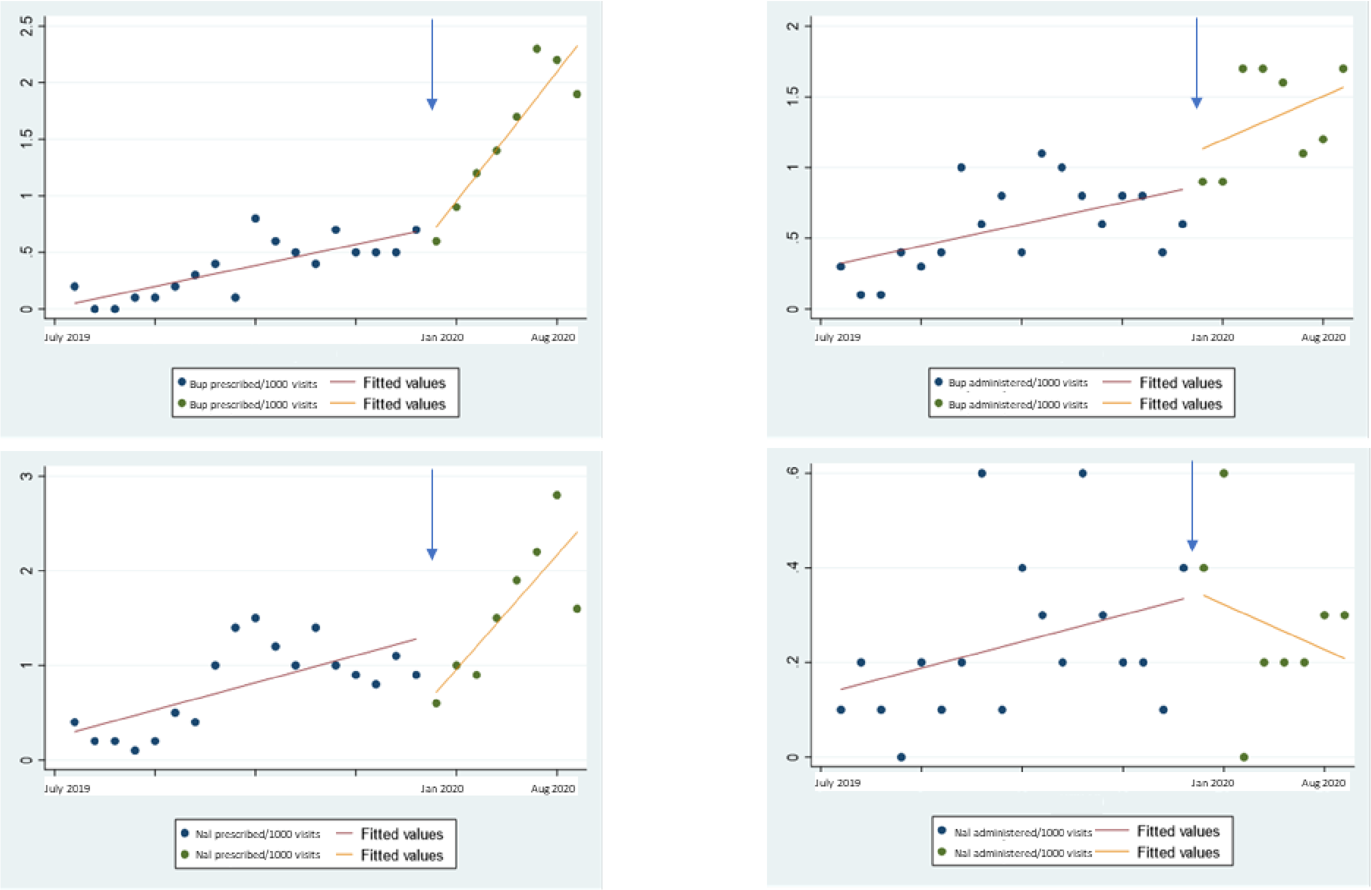

Supplement: Supplementary file 1 [file healthcare-10-02393-s001.zip › healthcare-1920689-supplementary.pdf]
